# Prognostic role of tumor necrosis in patients undergoing curative resection for gastric gastrointestinal stromal tumor: a multicenter analysis of 740 cases in China

**DOI:** 10.1002/cam4.1229

**Published:** 2017-10-23

**Authors:** Xuechao Liu, Haibo Qiu, Peng Zhang, Xingyu Feng, Tao Chen, Yong Li, Kaixiong Tao, Guoxin Li, Xiaowei Sun, Zhiwei Zhou, China Gastrointestinal Stromal Tumor Study Group (CN‐GIST)

**Affiliations:** ^1^ Sun Yat‐sen University Cancer Center State Key Laboratory of Oncology in South China Collaborative Innovation Center for Cancer Medicine Guangzhou 510060 China; ^2^ Department of Gastric Surgery Sun Yat‐sen University Cancer Center Guangzhou China; ^3^ Department of General Surgery Union Hospital Tongji Medical College Huazhong University of Science and Technology Wuhan China; ^4^ Department of General Surgery Guangdong General Hospital Guangzhou China; ^5^ Department of General Surgery Southern Medical University Nanfang Hospital Guangzhou China

**Keywords:** Gastrointestinal stromal tumor, nuclear atypia, prognosis, tumor necrosis

## Abstract

Tumor necrosis is associated with poor clinical outcomes in many malignancies. We aimed to determine whether tumor necrosis was an independent predictor of outcomes in gastric gastrointestinal stromal tumors (GISTs). We retrospectively analyzed data from 740 patients undergoing curative resection for gastric GIST at four centers between 2001 and 2015. Disease‐free survival (DFS) was estimated with the Kaplan–Meier method, and associations with prognosis were assessed with Cox regression models. Tumor necrosis was present in 122 cases (16.5%). The prevalence of tumor necrosis increased with higher risk‐stratification, including 0.7%, 7.4%, 17.3%, and 39.3% for very low‐, low‐, intermediate‐ and high‐risk tumors, respectively (*P* < 0.001). Tumor necrosis was associated with aggressive tumor biology, such as larger tumor size, higher mitotic index, tumor rupture, and presence of nuclear atypia (all *P* < 0.05). Multivariate analysis revealed that tumor necrosis was an independent predictor of unfavorable DFS (HR: 2.641; 95% CI: 1.359–5.131; *P* = 0.004). When stratified by the modified National Institutes of Health (NIH) classification, tumor necrosis still independently predicted DFS in high‐risk patients (*P* = 0.001) but not in non‐high‐risk patients (*P* = 0.349). The 5‐year DFS rate in high‐risk patients with and without tumor necrosis was 56.5% and 82.9%, respectively (*P* = 0.004). Notably, the prognostic significance of tumor necrosis was maintained when the patients were stratified by age, sex, tumor location, tumor size, and mitotic index (All *P* < 0.05). Tumor necrosis is a useful predictor of outcomes in gastric GIST, especially in high‐risk patients. Based on these results, we recommend that the current NIH classification should be further improved and expanded to include tumor necrosis as a valuable prognostic indicator.

## Introduction

Gastrointestinal (GI) stromal tumors (GISTs) are the most common mesenchymal tumors in the gastrointestinal tract, which is known to be refractory to conventional chemoradiotherapy 1, 2. Although they can arise throughout the entire GI tract, most of these tumors (60–70%) are commonly found in the stomach 3. GISTs are characterized by mutations of KIT or platelet‐derived growth factor receptor alpha (PDGFRA), which provides the rationale for benefiting from targeted therapy 4, 5, 6. Due to their sensitivity to selective tyrosine kinase inhibitors (TKIs), GISTs have recently gained considerable attention 7, 8, 9. Unfortunately, tumor recurrence is common after complete surgical resection, which usually occurs in the liver and/or peritoneum 10. Therefore, identifying independent prognostic factors is important for individualized risk stratification, tailored follow‐up protocols and evidence‐based counseling for postoperative treatment options.

Therefore, several risk‐prediction schemes have been proposed, including the National Institutes of Health (NIH) classification and modified NIH classification and the Armed Forces Institute of Pathology (AFIP) classification 11, 12. The modified NIH classification, which incorporates the established independent risk factors of tumor size, mitosis count, tumor site and tumor rupture, was best at identifying a single subgroup of patients with poor prognosis 13, 14. However, the prognosis for patients with GISTs can vary, even when they have the same risk‐stratification. Thus, clinicians have been seeking other prognostic factors that may help to precisely calculate the risk of recurrence. In recent years, tumor necrosis, one of the most potentially attractive histological prognosticators, has been evaluated as a prognostic biomarker in many malignancies 15, 16, 17. However, in GIST, the relationship between tumor necrosis and prognosis remains less clear. Novitsky et al. studied the survival of 50 patients with gastric GISTs, of which 22% demonstrated tumor necrosis. In their analysis, the presence of tumor necrosis was statistically associated with tumor recurrence 18. However, Lv et al. reported, based on a series of 114 primary GIST patients, that tumor necrosis was not an independent predictor of poor outcomes by multivariate analysis 19.

Considering the limited number of patients in previous studies, we performed a large‐scale multicenter retrospective analysis to determine the clinical utility of tumor necrosis in patients undergoing curative resection for gastric GIST.

## Material and Methods

### Study population

A total of four medical centers in China provided data for the current analysis: Sun Yat‐sen University Cancer Center, The Union Hospital Huazhong University of Science and Technology, Southern Medical University Nanfang Hospital and Guangdong General Hospital. After combining the data sets, reports were generated to solve data inconsistencies and some data‐integrity problems by personal correspondence. Finally, our study population comprised 740 patients undergoing curative resection for gastric GIST between 2001 and 2015.

Clinical, pathological, and survival data were collected for each patient, including age, sex, histological subtype, postoperative tumor characteristics, and survival duration. All patients were histologically confirmed for the presence of gastric GIST according to standard pathologic procedures. Patients with other synchronous malignancies or with incomplete clinicopathological and follow‐up data were excluded from the study. Patients undergoing neoadjuvant imatinib or chemoradiotherapy were also excluded.

### Pathologic evaluation

The routine pathologic assessment of GIST specimens was based on a minimum of three formalin‐fixed, paraffin‐embedded tissue blocks per tumor. Tumor necrosis was classified as coagulative, liquefactive or hyalinizing. Coagulative necrosis was the most common form or tumor necrosis in GIST cases, characterized by homogeneous clusters and sheets of degenerating and dead cells. In accordance with previous studies, tumor necrosis was defined as the presence of microscopic coagulative necrosis lacking inflammation or fibrosis, regardless of the ratio of tumor necrosis and tumor cells, whereas the presence of necrosis on gross examination was disregarded 15, 20, 21. An additional parameter was recorded in the cases: Nuclear atypia was defined by nuclear enlargement, hyperchromasia, pleomorphism, increased nucleocytoplasmic ratio, or vesicular nature of the chromatin.

### Patient follow‐up

The patients were followed up routinely after surgery—annually for very low‐ or low‐risk patients and every 6 months for intermediate‐ or high‐risk patients. The follow‐up assessment consisted of medical history, physical examination, routine laboratory testing, endoscopy, and dynamic abdominal pelvic computerized tomography scan. The latest follow‐up date for the study was in February 2016. Disease‐free survival (DFS) was defined as the time from surgery to the first event of either recurrent disease or death.

### Statistical analysis

Statistical analyses were performed by SPSS version 19.0 (IBM Corporation, Armonk, NY, USA). The results were reported as the mean with 95% confidence intervals (CI). Comparisons among categorical variables were performed using chi‐square test. The probability of survival was estimated using the Kaplan–Meier method, and significant differences were analyzed by log‐rank test. The variables that were significant (*P* < 0.05) in the univariate analysis were included in a final multivariate Cox proportional hazards model. All tests were two‐sided, and statistical significance was set at *P* < 0.05. All data in our study have been recorded at Sun Yat‐sen University Cancer Center for future reference (number RDDA2017000252).

## Results

**Figure 1:**
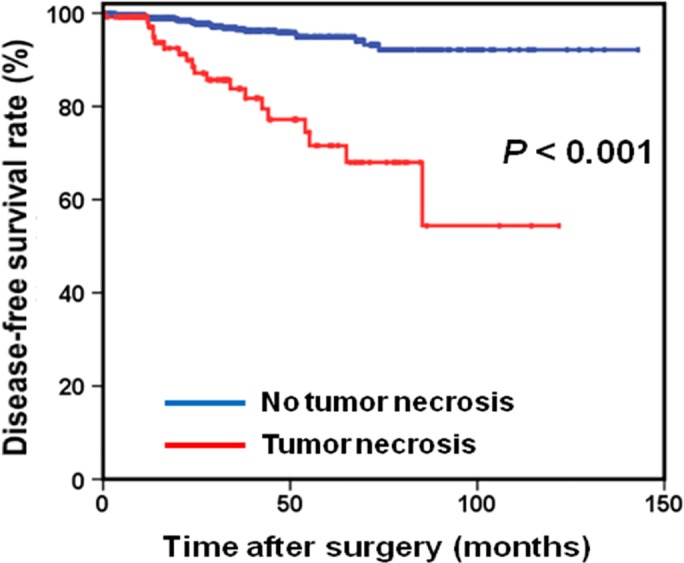
Disease‐free survival based on tumor necrosis in patients with gastric gastrointestinal stromal tumor.

Among the 740 patients studied, 368 (49.7%) were men and 372 (50.3%) were women, with a median age of 59 years (range, 20–91 years) (Table 1). According to the modified NIH classification, there were 147 (19.9%) very low‐risk, 242 (32.7%) low‐risk, 160 (21.6%) intermediate‐risk, and 191 (25.8%) high‐risk patients. Tumor necrosis, present in 122 patients (16.5%), was significantly associated with higher risk stratification, including 0.7%, 7.4%, 17.3%, and 39.3% for very low‐, low‐, intermediate‐ and high‐risk tumors, respectively (*P* < 0.001). In addition, nuclear atypia was present in 63 patients (9.6%). The median follow‐up period was 33.2 months (ranging from 1 to 143 months). The 5‐year DFS rate in patients with and without tumor necrosis was 71.6% and 94.9%, respectively (*P* < 0.001) (Fig. 1).

**Table 1:** Clinicopathologic characteristics of patients with gastric gastrointestinal stromal tumors

	No. of patients (%)
Sex	
Male	368 (49.7)
Female	372 (50.3)
Age (years)	
<60	373 (50.4)
≥60	367 (49.6)
Tumor size (cm)	
≤2	162 (21.9)
>2 ≤ 5	313 (42.3)
>5 ≤ 10	190 (25.7)
>10	75 (10.1)
Mitotic index (/50 HPF)	
<5	538 (72.7)
≥5 ≤ 10	13 (15.3)
>10	89 (12.0)
Tumor location	
Upper third	349 (47.2)
Middle third	296 (40.0)
Lower third	95 (12.8)
Histological subtype	
Spindle type	667 (90.1)
Epithelioid type	21 (2.8)
Mixed type	52 (7.0)
Tumor rupture	
No	735 (99.32)
Yes	5 (0.7)
Nuclear atypia	
No	594 (90.4)
Yes	63 (9.6)
Tumor necrosis	
No	618 (83.5)
Yes	122 (16.5)
Postoperative imatinib	
No	551 (74.5)
Yes	189 (25.5)

The relationships of tumor necrosis with clinicopathological features are shown in Table 2. The presence of tumor necrosis was significantly associated with larger tumor size (*P* < 0.001), higher mitotic index (*P* < 0.001), tumor rupture (*P* = 0.009), and presence of nuclear atypia (*P* < 0.001). However, it was not associated with age, sex, tumor location or histological subtype.

**Table 2:** Relationship between clinicopathological characteristics and tumor necrosis

	No tumor necrosis	Tumor necrosis	*P*‐value
	(*n* = 618)	(*n* = 122)	
Sex			0.210
Male	301	67	
Female	317	55	
Age (years)			0.276
<60	317	56	
≥60	301	66	
Tumor size (cm)			<0.001
≤5	443	32	
>5	175	90	
Mitotic index (/50 HPF)			<0.001
<5	480	58	
≥5	138	64	
Tumor location			0.058
Upper third	301	48	
Middle/lower third	317	74	
Histological subtype			0.295
Spindle type	561	107	
Epithelioid/mixed type	57	15	
Tumor rupture			0.009
No	616	119	
Yes	2	3	
Nuclear atypia			<0.001
No	519	75	
Yes	37	26	

Our univariate analysis showed that tumor size, mitotic index, and tumor necrosis were all associated with DFS. However, in multivariate analysis, only tumor necrosis (HR: 2.641; 95% CI: 1.359–5.131; *P* = 0.004), tumor size (HR: 2.623; 95% CI: 1.189–5.783; *P* = 0.017) and mitotic index (HR: 3.057; 95% CI: 1.557–6.013; *P* = 0.001) were independently associated with DFS (Table 3).

**Table 3:** Univariate and multivariate analyses of all patients for disease‐free survival

	Univariate analysis		Multivariate analysis	
	HR (95% CI)	*P*‐value	HR (95% CI)	*P*‐value
Sex		0.575		
Male	1.00			
Female	0.841 (0.458, 1.542)			
Age (years)		0.640		
<60	1.00			
≥60	1.156 (0.630, 2.123)			
Tumor size (cm)		<0.001		0.017
≤5	1.00		1.00	
>5	5.740 (2.822, 11.678)		2.623 (1.189, 5.783)	
Mitotic index (/50 HPF)		<0.001		0.001
<5	1.00		1.00	
≥5	5.055 (2.724, 9.378)		3.057 (1.557, 6.013)	
Tumor location		0.071		
Upper third	1.00			
Middle/lower third	1.806 (0.951, 3.430)			
Histological subtype		0.518		
Spindle type	1.00			
Epithelioid/mixed type	1.330 (0.560, 3.158)			
Tumor rupture		0.009		0.237
No	1.00		1.00	
Yes	6.752 (1.628, 28.012)		2.637 (0.528, 13.161)	
Nuclear atypia				
No	1.00			
Yes	1.873 (0.719, 4.877)			
Tumor necrosis		<0.001		0.004
No	1.00		1.00	
Yes	5.703 (3.109, 10.463)		2.641 (1.359, 5.131)	
Postoperative imatinib		<0.001		0.151
No	1.00		1.00	
Yes	3.439 (1.819, 6.503)		1.670 (0.829, 3.363)	

**Figure 2:**
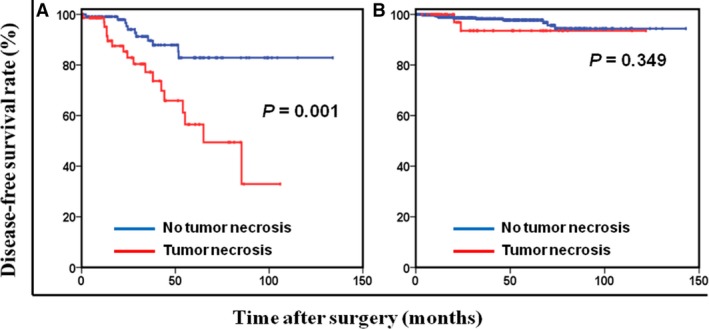
Disease‐free survival based on tumor necrosis in high‐risk (A) and non‐high‐risk (B) patients.

**Figure 3:**
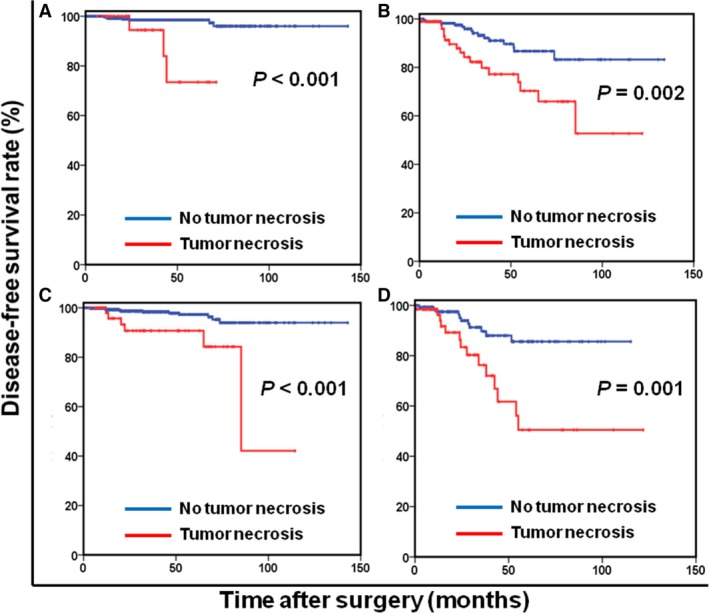
Disease‐free survival based on tumor necrosis in patients with tumor size ≤ 5 cm (A), tumor size > 5 cm (B), mitotic index ≤ 5/50 HPF (C), and mitotic index > 5/50 HPF (D).

When patients were stratified by modified NIH classification, tumor necrosis was present in 39.3% and 8.6% of high‐risk and non‐high‐risk patients, respectively. We found that the prognostic significance of tumor necrosis was maintained in high‐risk patients (*P* = 0.001; Fig. 2A) but not in non‐high‐risk patients (*P* = 0.349; Fig. 2B). High‐risk patients with tumor necrosis had a significantly poorer 5‐year DFS rate than did those without tumor necrosis (56.5% vs. 82.9%, respectively; *P* = 0.004). Notably, tumor necrosis was still independently associated with DFS stratified by age, sex, tumor location, mitotic index, and tumor size (All *P* < 0.05; Fig. 3). Furthermore, 113 high‐risk patients (59.2%) received adjuvant imatinib therapy, with the median duration of therapy was 22.4 months (range, 1.3–62.8 months). Among high‐risk patients with tumor necrosis, those with adjuvant imatinib therapy had a significantly higher 5‐year DFS rate than those without adjuvant imatinib therapy (71.1% vs. 43.1%, respectively; *P* = 0.026; Figure S1).

## Discussion

With the advent of patient‐tailored targeted treatment, identifying independent prognostic factors for more accurate risk stratification and monitoring treatment has become more important. Our current study confirmed that the presence of tumor necrosis, as part of the pathological findings, independently predicts DFS in patients undergoing curative resection for gastric GIST, especially in high‐risk patients. It might enable clinicians to generate more accurate prognostic prediction, determine individual treatment strategies and plan standard follow‐up protocols.

Tumor necrosis, a distinct type of cell death, is usually associated with abnormal processes, such as exposure to various toxins or teratogens, infections, trauma, and ischemia 22, 23. However, until now, the mechanisms by which tumor necrosis results in poor prognosis were unclear and poorly understood. A potential hypothesis is that rapid cell proliferation outgrowing the vasculature leads to hypoxic conditions in the tumor, resulting in subsequent tumor cell death and promoting the metastatic cascade 24, 25, 26. Additionally, tumor necrosis is directly associated with an attenuation of the local infiltration of inflammatory cells and the presence of systemic inflammatory response. It has been speculated that the combination of inflammation and necrosis may generate an environment to stimulate angiogenesis, cell proliferation and cancer progression 27, 28.

In recent years, tumor necrosis has been established as a potential prognostic marker for a variety of malignancies, including lung cancer, renal cell carcinoma, pancreatic cancer, colorectal cancer and GIST. A recent systematic review of 20 published studies demonstrated tumor necrosis as an independent and significant prognostic factor, which could affect therapeutic decisions in patients with non‐small cell lung carcinoma 16. Sengupta et al. reported, in a series of 3009 renal cell carcinomas (RCCs), that coagulative tumor necrosis was retained as an independent prognostic marker for clear cell and chromophobe RCC and suggested that it to be incorporated into current prognostic models for more accurate risk estimation 29.

Likewise, Hiraoka et al. evaluated and reported the prognostic significance of tumor necrosis is 348 patients with pancreatic ductal carcinoma (PDC). They found that tumor necrosis was associated with aggressive pathologic characteristics and found it to be a simple, accurate, and reproducible predictor of poor outcomes in PDC patients 30. However, the literature regarding the relationship between tumor necrosis and GIST prognosis has been inconclusive. A recent study on 67 patients undergoing surgical resection for GIST showed that tumor necrosis was independently associated with DFS 31. Gouveia et al. reported that tumor necrosis was limited as a predictor of disease‐specific survival 32. Finally, another recent study from Korea also suggested that tumor necrosis was not an independent predictor of clinical outcomes for GIST patients 33.

In this study, tumor necrosis was observed in 16.5% of gastric GIST patients, which was consistent with previously published studies 33, 34. In line with previous studies, we found that gastric GIST patients with tumor necrosis had significantly shorter DFS than the patients without tumor necrosis. We also observed that DFS in gastric GIST was independently associated with only three of the analyzed variables: tumor necrosis, tumor size and mitotic index. Furthermore, tumor necrosis was associated with larger tumor size, higher mitotic index, tumor rupture and presence of nuclear atypia. These results strongly support the findings of previous studies, in which tumor necrosis significantly paralleled tumor progression and more aggressive tumor biological behavior 35, 36. However, tumor rupture lacked independent prognostic significance in multivariate analysis, although they have been recognized as important prognostic factors by the current National Comprehensive Cancer Network Clinical Practice Guidelines. We speculate that tumor necrosis might exhibit a more potent prognostic value than that of tumor rupture.

Notably, the prognostic significance of tumor necrosis was only maintained in high‐risk patients, but not in non‐high‐risk patients. These data suggest that in clinical practice, tumor necrosis may complement the modified NIH classification in identifying “very high risk” patients. In addition, we found that high‐risk patients with tumor necrosis significantly benefited from adjuvant imatinib therapy. However, whether high‐risk patients with tumor necrosis are likely to benefit from a prolonged or lifelong imatinib treatment is also of considerable interest 37. Future studies, especially large‐scale prospective randomized controlled studies, are warranted to validate these notions.

Thisstudy has several limitations. As with all retrospective studies, first and foremost, there are limitations in this study inherent to retrospective study design and data collection. To maintain homogeneity of the study population, we excluded patients with neoadjuvant imatinib treatment, which may have resulted in selection bias. Furthermore, we used DFS as the primary outcome, and our conclusion may have been strengthened with additional survival measures, such as overall survival. Finally, our study lacked information on the extent of tumor necrosis, which may have influenced the results.

## Conclusions

Tumor necrosis is independently associated with DFS in gastric GIST, especially in high‐risk patients. As a simple, accurate, and reproducible prognostic indicator, the presence of tumor necrosis should be routinely reported in pathological assessment for consideration in patient counseling and treatment decision‐making.

## Conflict of Interest

The authors report no conflicts of interest in this work.

## Supporting information


**Figure S1.** Disease‐free survival based on adjuvant imatinib therapy in high‐risk patients with tumor necrosis.
