# Supplementary figures and images for: Prognostic role of tumor necrosis in patients undergoing curative resection for gastric gastrointestinal stromal tumor: a multicenter analysis of 740 cases in China
Source: Cancer Med. 2017 Oct 23;6(12):2796–803. doi: 10.1002/cam4.1229 (PMC5727342; doi:10.1002/cam4.1229)

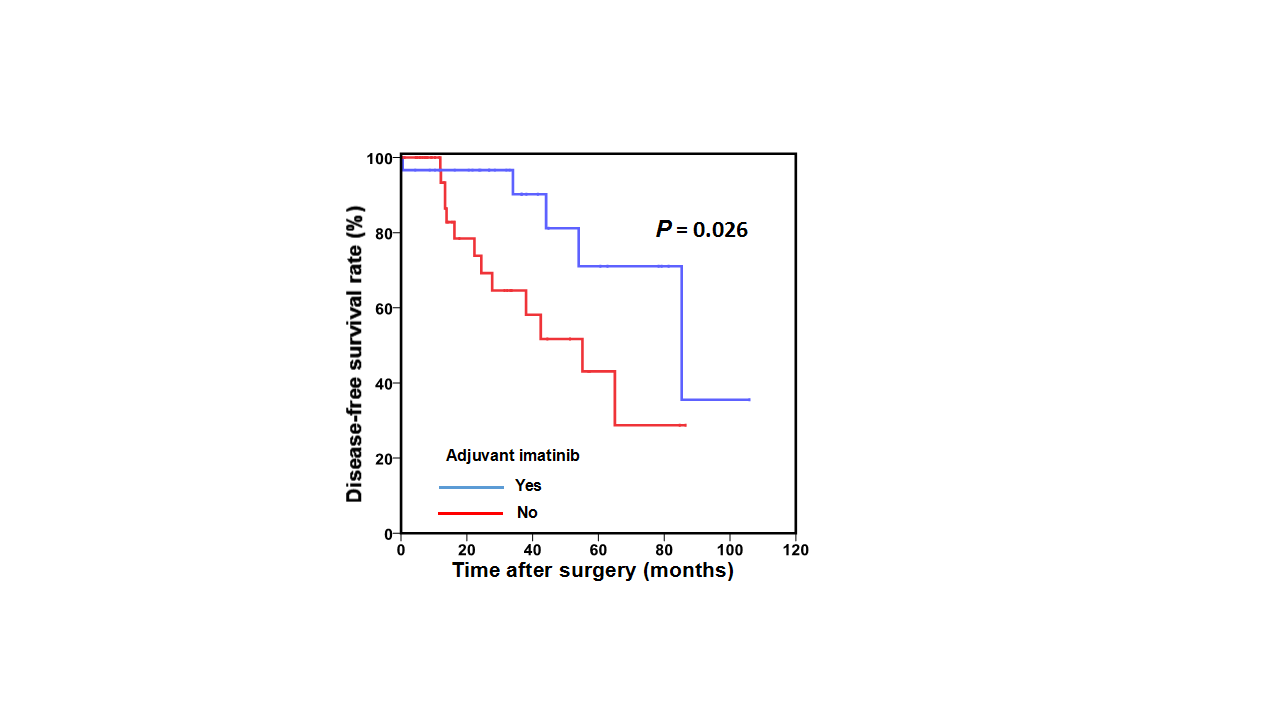

Supplement: Supplementary file 1 — Figure S1. Disease‐free survival based on adjuvant imatinib therapy in high‐risk patients with tumor necrosis. [file CAM4-6-2796-s001.tiff]
